# Risk Estimation of Infectious and Inflammatory Disorders in Hospitalized Patients With Acute Ischemic Stroke Using Clinical-Lab Nomogram

**DOI:** 10.3389/fneur.2021.710144

**Published:** 2021-12-10

**Authors:** Junhong Li, Jingjing Huang, Tingting Pang, Zikun Chen, Jing Li, Lin Wu, Yuqiang Hu, Wei Chen

**Affiliations:** ^1^Department of Neurology, The First Affiliated Hospital of Guangxi University of Chinese Medicine, Nanning, China; ^2^Guangxi University of Chinese Medicine, Nanning, China

**Keywords:** stroke, infection, inflammation, nomogram, hospitalized patients

## Abstract

**Background:** Infections after acute ischemic stroke are common and likely to complicate the clinical course and negatively affect patient outcomes. Despite the development of various risk factors and predictive models for infectious and inflammatory disorders (IAID) after stroke, more objective and easily obtainable predictors remain necessary. This study involves the development and validation of an accessible, accurate nomogram for predicting in-hospital IAID in patients with acute ischemic stroke (AIS).

**Methods:** A retrospective cohort of 2,257 patients with AIS confirmed by neurological examination and radiography was assessed. The International Statistical Classification of Diseases and Health related Problem's definition was used for IAID. Data was obtained from two hospitals between January 2016 and March 2020.

**Results:** The incidence of IAID was 19.8 and 20.8% in the derivation and validation cohorts, respectively. Using an absolute shrinkage and selection operator (LASSO) algorithm, four biochemical blood predictors and four clinical indicators were optimized from fifty-five features. Using a multivariable analysis, four predictors, namely age (adjusted odds ratio, 1.05; 95% confidence interval [CI], 1.038–1.062; *p* < 0.001), comatose state (28.033[4.706–536.403], *p* = 0.002), diabetes (0.417[0.27–0.649], *p* < 0.001), and congestive heart failure (CHF) (5.488[2.451–12.912], *p* < 0.001) were found to be risk factors for IAID. Furthermore, neutrophil, monocyte, hemoglobin, and high-sensitivity C-reactive protein were also found to be independently associated with IAID. Consequently, a reliable clinical-lab nomogram was constructed to predict IAID in our study (C-index value = 0.83). The results of the ROC analysis were consistent with the calibration curve analysis. The decision curve demonstrated that the clinical-lab model added more net benefit than either the lab-score or clinical models in differentiating IAID from AIS patients.

**Conclusions:** The clinical-lab nomogram predicted IAID in patients with acute ischemic stroke. As a result, this nomogram can be used for identification of high-risk patients and to further guide clinical decisions.

## Introduction

Infectious and inflammatory disorders (IAID), such as pneumonia and urinary tract infection, are common complications of acute ischemic stroke (AIS), with high rates of up to 30–65% (1, 2). Growing evidence suggests that IAID increases the occurrence of oropharyngeal dysphagia, impaired consciousness, hemorrhagic transformation, stroke severity, mortality, and stroke outcome (3, 4). Systemic inflammatory response and immune dysregulations after stroke may play an essential role in brain injury and recovery (5, 6). It means that IAID may contribute to stroke risk and adverse outcomes after stroke through various potential pathways (7). Moreover, early lipid-lowering agent use appears to be associated with an increased risk of post-stroke infection (6). The optimal treatment for IAID during AIS is a source of considerable uncertainty, creating a dilemma for clinicians.

On the one hand, the preventive overuse of antibiotics will diminish antimicrobial efficacies against numerous multidrug-resistant bacterial pathogens. On the other hand, however, the delayed treatments of IAID will likely result in adverse outcomes. Therefore, it is necessary to screen vulnerable patients by identifying early predictors of post-stroke infection, thereby carrying out early warning and implementing tailored preventive strategies.

Early studies have identified several risk factors for IAID: older age, male sex, diabetes mellitus, congestive heart failure, hypertension, atrial fibrillation, chronic obstructive pulmonary disease, dysphagia, and stroke severity (8, 9). Recent studies showed that stroke-induced suppression of the innate and adaptive immune system is another major cause of infections in patients with stroke (10). The immunosuppression decreases in peripheral blood lymphocyte count or increases in the neutrophil-to-lymphocyte ratio (11, 12). China is often considered to have a high incidence of nosocomial infection, and there is not enough research in this area (13). Besides, the low sensitivity of sputum cultures makes it hard to diagnose IAID (11, 14). Thus, a more objective and easily obtainable predictor is needed in routine clinical practice.

This study aimed to evaluate the predictors and develop and validate an easily accurate nomogram for predicting in-hospital IAID after AIS and calculate probabilistic estimates to guide clinical decisions.

## Methods

### Study Design

The ethics committee approved the study (The First Affiliated Hospital of Guangxi University of Chinese Medicine, 2020-029-01); ethical approval was given for this anonymous retrospective study conducted under the Declaration of Helsinki.

The study was authorized by the Institutional Review Board and the local ethics committee. However, the written informed consent was waived from all patients due to the study's retrospective nature.

### Eligibility Criteria

This retrospectively designed observational study evaluated the institutional database for medical records from two hospitals (The First Affiliated Hospital of Guangxi University of Chinese Medicine and its branches) between January 2016 and March 2020. Acute Ischaemic Stroke (AIS) was confirmed by neurological examination and radiography, in accordance with WHO (15) (World Health Organization). Patients who were 18 years or older were enrolled in our study if the baseline cranial CT or MRI was performed within 24 hours of symptom onset or patients with acute ischemic stroke who were admitted within 7 days of symptom onset were recruited into the study (*n* = 2,273). Patients whose blood biomarkers were analyzed within 7 days of onset of symptoms and whose complete data were available; were included. Patients who met the following conditions were then excluded: (1) Patients with an active infection monitored in an intensive care unit (*n* = 6), (2) tumor or aneurysm presumed to be the potential cause of infections (*n* = 3), (3) hemorrhagic transformation of AIS (*n* = 5), (4) history of pyrexia or active infection in the last 2 weeks, and (5) surgical intervention within three months (*n* = 2). Finally, a total of 2,257 patients were analyzed.

**Figure 1:**
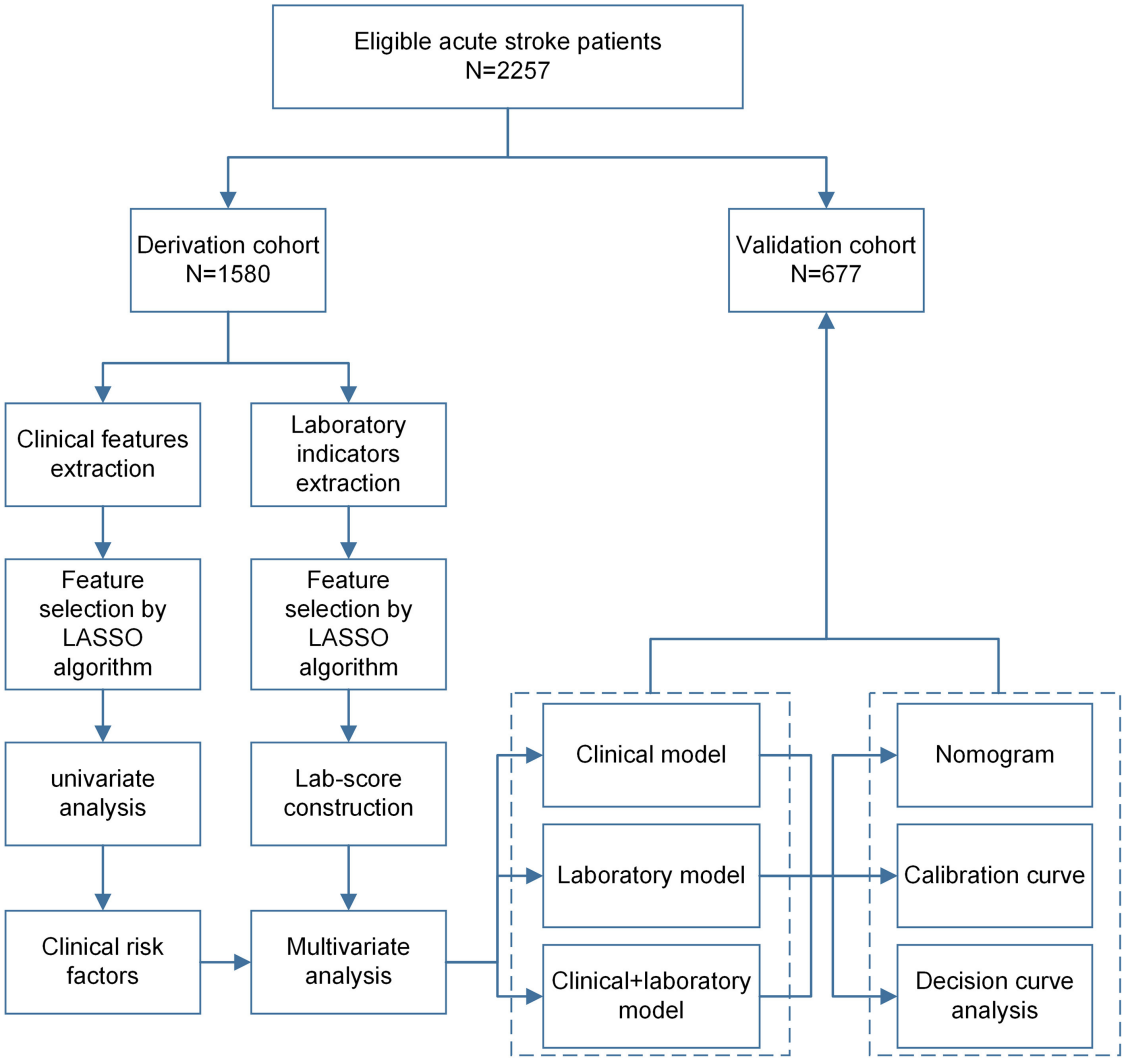
The workflow of this study. LASSO, least absolute shrinkage and selection operator.

This is a retrospective cohort study, and eligible patients were randomly divided into derivation cohort (70%, 1,579) and validation cohort (30%, 678) using the sample function in R version 3.6.1 software (https://www.r-project.org/). International Statistical Classification of Diseases and Related Health Problems Tenth Revision (ICD-10; 2019 version; I63 Cerebral Infarction) (16). Diagnosis codes were used to identify whether individuals had been diagnosed with infections or inflammatory disorders during hospitalization (17). However, regardless of the number of diagnosis codes, these individuals were considered exposed, as previously reported (18, 19). Figure 1 presents the workflow of this analysis, including the derivation and validation sections.

### Outcome Definition

Post-stroke infection was defined as any infection transpiring within 7 days after the onset of stroke. Definitions of infections were based on the Centers for Disease Control and Prevention (CDC) criteria published in 1988 (20). We differentiated infections between pneumonia, urinary tract infection, and 'other infections'. UTI was described as the presence of suitable clinical symptoms and/or signs with positive microbiological cultures, or negative cultures with leukocytosis, and/or fever. Pneumonia was defined as the presence of positive respiratory clinical symptoms and/or signs, with at least one of the following: leukocytosis (>11 × 109 cells/L), fever (temperature ≥ 38.0 C), or a positive chest radiograph. The “other infections” group included patients with fever combined with leukocytosis who did not meet the pneumonia or UTI diagnosis criteria (21, 22).

The influx of peripheral leukocytes into the cerebral parenchyma and activation of endogenous microglia subsequently after focal cerebral ischemia indicate a vigorous inflammatory reaction (23). Secondary damage develops as a repercussion of brain edema, vasomotor/hemodynamic deficits, and post-ischemic microvascular stasis. This elicits hypoperfusion and post-ischemic inflammation, thus entailing activation of microglia and brain infiltration of peripheral inflammatory cells (24).

### Candidate Predictors Acquisition

Candidate predictors include clinical and baseline laboratory variables such as age, gender, nationality. In addition, Charlson comorbidity index (CCI) (25), hemiplegia, speech impediment, comatose state, dyslipidemia, hypertension, atrial fibrillation, coronary heart disease (CHD), peripheral vascular diseases, cerebrovascular diseases (CVD), chronic obstructive pulmonary disease (COPD), hepatitis, diabetes mellitus (DM), congestive heart failure (CHF) were noted. Also, current smoking, current drinking, systolic blood pressure (SBP), diastolic blood pressure (DBP), white blood cell (WBC), neutrophils, percentage of neutrophils, lymphocyte, percentage of lymphocyte, monocyte, percentage of monocyte, red blood cell (RBC), hematocrit (HCT), mean corpuscular hemoglobin concentration (MCHC), mean corpuscular hemoglobin (MCH), platelet, D-dimer, C-reactive protein (CRP), procalcitonin (PCT), and hemoglobin (HGB) were considered. All these variables were assessed and recorded on admission.

### Predictor Selection

We used the least absolute shrinkage and selection operator (LASSO) algorithms implemented in R package glmnet to select relatively fewer optimized features and then built a regression model including selected variates (26). Notably, the LASSO algorithm yields a model with parsimonious sets of features for discriminating IAID and non-IAID patients with AIS. However, the overfitting pursues a slight deviation, which makes the model partial to complexity (26, 27). The deviation between the fitted data distribution and the actual distribution is slight, but the variance and the absolute value of the slope of the curve are substantial. Therefore, LASSO reduces the coefficients of features with less importance to zero by setting penalty operators, and only non-zero coefficients (important features) are retained (28). The non-zero coefficient of the selected feature is defined as lab-score. The lab score was calculated for each patient of selected features that were weighted by their respective coefficients.

### Statistical Analysis

Subsequent analysis was performed using R version 3.6.1. Statistical comparisons between the IAID group and the non-IAID group were performed by independent-samples *t*-test, Chi-square test, or Mann-Whitney U test, where appropriate. Variables with *p* < 0.10 in the results of the univariate analysis were considered as potential confounding factors. Independent predictors of in-hospital IAID after AIS were screened using multivariable logistic regression. The glmnet (29), rms (30), pROC (31), CalibrationCurves (32), and rmda (33, 34) packages were used for LASSO logistic regression model, IAID prediction nomogram, receiver operating characteristic (ROC) curve, calibration curve, and decision curve analysis (DCA), respectively. All statistical tests were two-sided, and *a p*-value < 0.05 was considered significant.

## Results

### Clinical Characteristics

Table 1 summarizes the patient demographics, stroke characteristics, and laboratory indicators between the IAID and non-IAID groups. In total, 2,257 patients (1,455 males and 802 females; mean age, 66.7 ± 12.4 years) were identified for the final analysis. Of these, IAID positivity was 313 (19.8%) and 141 (20.8%) in the derivation and validation cohorts, respectively. In the derivation cohort, age (*p* < 0.001), comatose state (*p* < 0.001), diabetes (*p* = 0.017), and CHF (*p* < 0.001) were significant predictors of AIS.

**Table 1:** Summary statistics of patient characteristics between the IAID and non-IAID group.

				**univariate analysis**			**multivariate analysis**		
	**All patients (*N* = 2,257)**	**non-IAID (*N* = 1,799)**	**IAID(*N* = 458)**	***p*-value**	**OR**	**95%CI**	***p*-value**	**OR**	**95%CI**
Gender:				0.586	0.932	0.722–1.202			
Female									
Male	1,455 (64.5%)	1,164 (64.7%)	291 (63.5%)						
nationality:				0.246	0.793	0.536–1.173			
Han	1,969 (87.2%)	1,562 (86.8%)	407 (88.9%)						
Others	288 (12.8%)	237 (13.2%)	51 (11.1%)						
Age:				<0.001	1.052	1.04–1.064	<0.001	1.05	1.038–1.062
<60 years	641 (28.4%)	583 (32.4%)	58 (12.7%)						
60–75 years	1,033 (45.8%)	830 (46.1%)	203 (44.3%)						
75+ years	583 (25.8%)	386 (21.5%)	197 (43.0%)						
CCI:				0.007	1.148	1.038–1.27			
Low	1,767 (78.3%)	1,446 (80.4%)	321 (70.1%)						
Medium	402 (17.8%)	297 (16.5%)	105 (22.9%)						
High	88 (3.90%)	56 (3.11%)	32 (6.99%)						
Hemiplegia	1,430 (63.4%)	1,166 (64.8%)	264 (57.6%)						
Coma	11 (0.49%)	2 (0.11%)	9 (1.97%)	0.002	28.474	3.49–232.284	0.02	28.033	4.706–536.403
Dyslipidemia	230 (10.2%)	193 (10.7%)	37 (8.08%)	0.22	0.752	0.477–1.186			
Hypertension	2,218 (98.3%)	1,769 (98.3%)	449 (98.0%)	0.857	0.92	0.37–2.287			
Atrial fibrillation	112 (4.96%)	68 (3.78%)	44 (9.61%)	0.026	2.627	1.648–4.188			
CHD	2,022 (89.6%)	1,619 (90.0%)	403 (88.0%)	0.388	0.839	0.563–1.25			
Peripheral vascular Diseases	58 (2.57%)	47 (2.61%)	11 (2.40%)	0.577	0.792	0.348–1.799			
CVD	22 (0.97%)	17 (0.94%)	5 (1.09%)	0.768	0.795	0.173–3.646			
COPD	34 (1.51%)	26 (1.45%)	8 (1.75%)	0.926	1.048	0.388–2.83			
Hepatitis	2,106 (93.3%)	1,688 (93.8%)	418 (91.3%)	0.114	0.687	0.431–1.095			
Diabetes	2,101 (93.1%)	1,698 (94.4%)	403 (88.0%)	<0.001	0.394	0.261–0.595	<0.001	0.417	0.27–0.649
CHF	41 (1.82%)	15 (0.83%)	26 (5.68%)	<0.001	7.095	3.216–15.651	<0.001	5.488	2.451–12.912
Smoke	1,244 (55.1%)	974 (54.1%)	270 (59.0%)	0.05	1.282	1.000–1.645			
Drink	1,180 (52.3%)	930 (51.7%)	250 (54.6%)	0.029	1.317	1.029–1.687			

*CCI, Charlson comorbidity index; CHD, coronary heart disease; CVD, cerebral vascular disease; COPD, chronic obstructive pulmonary disease; CHF, congestive heart failure; OR, Odds Ratio; CI, confidence interval*.

### Feature Selection and Laboratory Score Construction

**Figure 2:**
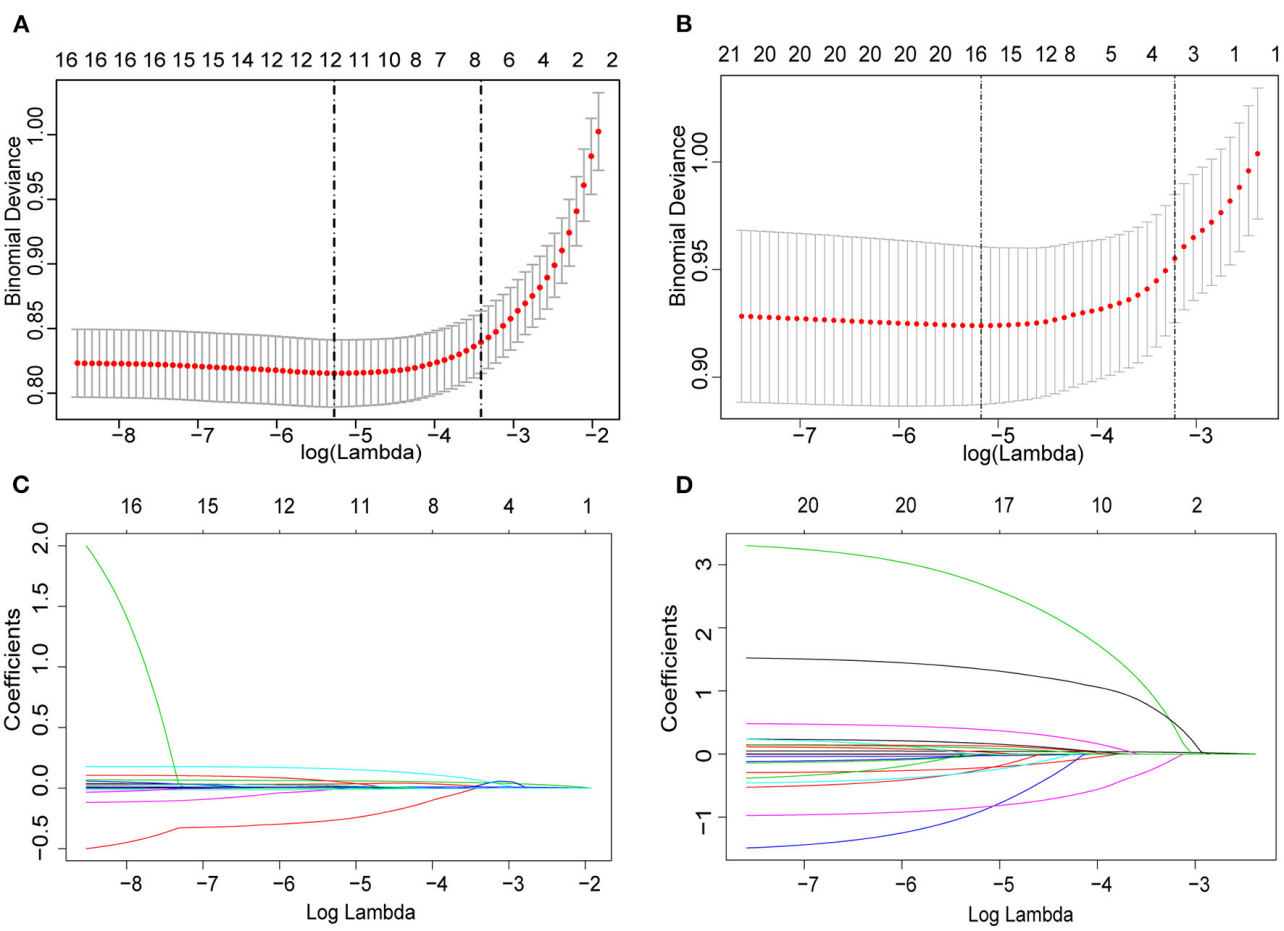
Predictor selection using the least absolute shrinkage and selection operator (LASSO) logistic regression model. **(A,B)** Identification of the optimal penalization coefficient lambda (λ) in the LASSO model. The dotted vertical line was plotted at the value selected using 10-fold cross-validation, for which the optimal λ resulted in 10 non-zero coefficients. **(C,D)** LASSO coefficient profiles of the 22 clinical features and 33 potential blood biochemical predictors. A coefficient profile plot was produced against the log (λ) sequence.

We first present the performance of the LASSO-based classifier on the derivation cohort, and fifty-five features were reduced to four clinical risk factors (Figures 2A,C) and four potential blood biochemical predictors (Figures 2B,D). A lab score was constructed using a multivariate regression model; then, four predictors were left, and their associated coefficients were calculated. The calculation formula was as follows: Lab-score=-6.73 + 0.07^*^neutrophil_percentage+0.17^*^monocyte_percentage-0.01^*^HGB+0.01^*^CRP.

### Development of a Clinical-Lab Model

**Figure 3:**
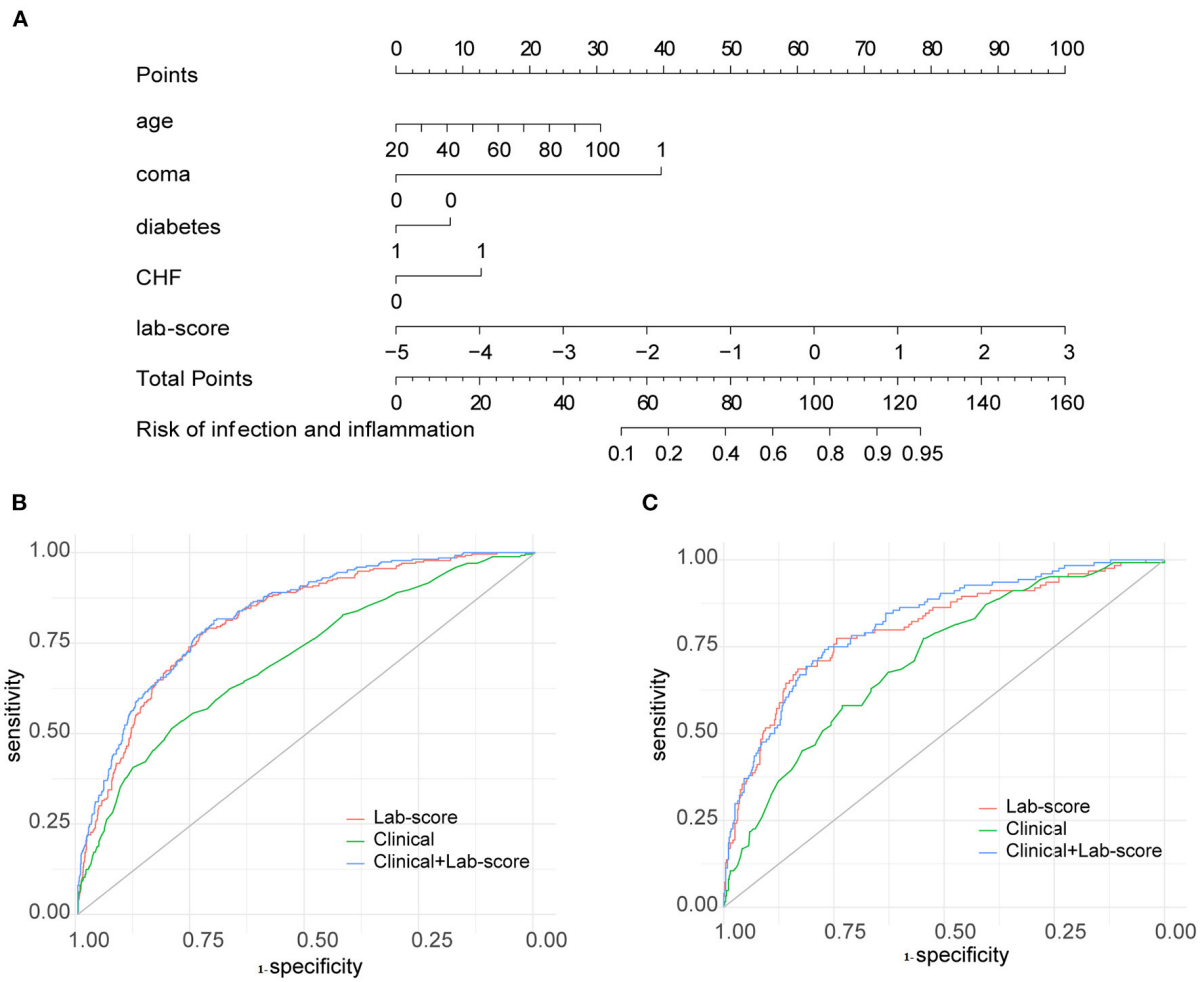
Developed clinical lab nomogram. **(A)** The clinical-lab nomogram was developed in the derivation cohort, with the lab score, age, coma, diabetes, CHF incorporated. Receiver operating characteristic (ROC) curve analyses of the clinical, lab-score, and clinical-lab models on hematoma expansion in the derivation cohort **(B)** and validation cohort **(C)**.

After feature selection by the LASSO algorithm, four clinical indicators were screened as the best subset of risk factors to develop the IAID risk model. In multivariate analysis, with results reported as odds ratio (95% confidence interval [CI]), age (1.05 [1.038–1.062]; *p* < 0.001), comatose state (no vs. yes; 2.8033 [4.706–5.36403]; *p* = 0.002), diabetes (no vs. yes; 0.417 [0.27–0.649]; *p* < 0.001), and CHF (no vs. yes; 5.488 [2.451–12.912]; *p* < 0.001) were independently associated with IAID (Table 1). Based on the above-mentioned independent predictors and lab score (2.718 [2.397–3.099]; *p* < 0.001), the clinical-lab model was developed (Table 1). The clinical-lab model incorporated with the above-mentioned independent predictors and lab-score (2.718 [2.397–3.099]; *p* < 0.001) was developed as shown in Table 1. Based on this model, a visualized clinical-lab nomogram was established for the risk estimation of IAID (Figure 3A). As shown in the nomogram, compared with other clinical risk factors, the lab score accounts for most of the scoring system, indicating a predominant role of quantitative parameters in predicting IAID.

### Diagnostic Performance of Clinical-Lab Model

**Figure 4:**
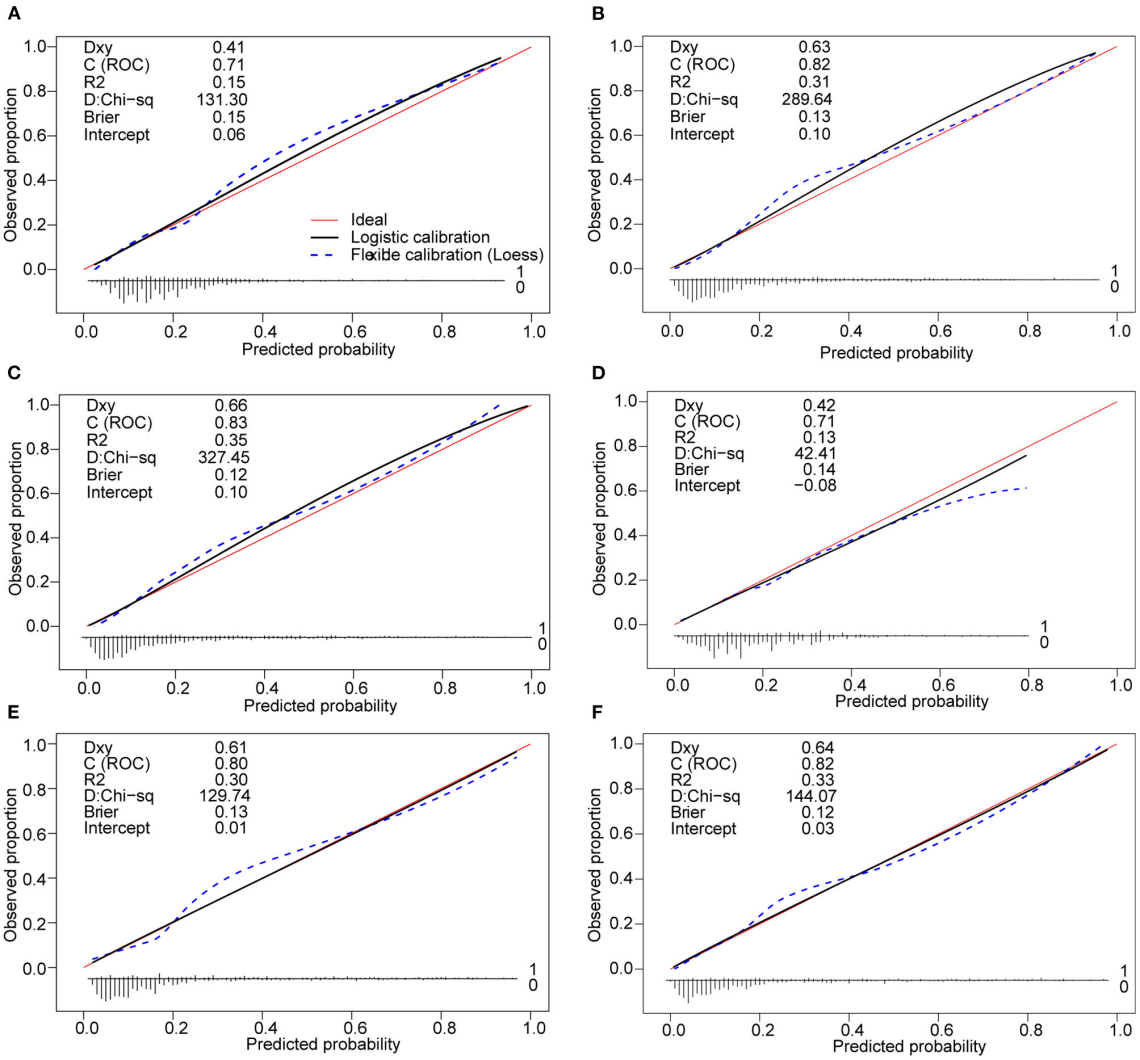
Calibration curves of the clinical nomogram and the model with the addition of lab-score in each cohort. **(A)** Calibration curve of the clinical nomogram in the derivation cohort. **(B)** Calibration curve of the lab-score nomogram in the derivation cohort. **(C)** Calibration curve of the clinical-lab nomogram in the derivation cohort. **(D)** Calibration curve of the clinical nomogram in the validation cohort t. **(E)** Calibration curve of the lab-score nomogram in the validation cohort. **(F)** Calibration curve of the clinical-lab nomogram in the validation cohort. Calibration curves depict the calibration of each model in terms of the agreement between the predicted risks of IAID and observed outcomes of IAID (IAID, infectious, and inflammatory disorders).

The clinical-lab model achieved satisfactory distinction between IAID-positive and IAID-negative patients, with an AUC of 0.828 (95%CI, 0.802–0.854; Figure 3B) in the derivation cohort and an AUC of 0.822 (95%CI, 0.766–0.875; Figure 3C, Table 2) in the validation cohort, indicating a good inter-observer agreement. Interestingly, in both cohorts, the clinical-lab model suggested the highest discrimination ability for IAID as compared with the lab-score model (0.828 vs. 0.812, *p* = 0.041; 0.822 vs. 0.806, *p* = 0.033; respectively; Figure 3B, Table 2) or the clinical model (0.828 vs. 0.706, p < 0.001; 0.822 vs. 0.716, p < 0.001; respectively; Figure 3C, Table 2). Compared with the lab-score or clinical model, the clinical-lab model had the highest sensitivity for detecting IAID in both datasets. The calibration curve of the clinical-lab model graphically showed good consistency between nomogram estimation and actual observation (Figures 4C,F, Table 2); The C-index values of the derivation group and the validation group were 0.83 vs. 0.82, respectively. Similarly, the same was true for the clinical and lab-score models via bootstrapping validation (Figures 4A,B,D,E, Table 2). The results of the ROC analysis were consistent with the calibration curve analysis, which proved the reliability of our analysis.

**Table 2:** Accuracy of the three models for predicting IAID.

	**AUC (95% CI)**	**Youden index**	**Sensitivity**	**Specificity**	**PPV**	**NPV**
**Derivation cohort**						
Clinical model	0.7064 (0.6711–0.7417)	0.309	0.513	0.796	0.4	0.86
Lab–score	0.8156 (0.7883–0.843)	0.508	0.788	0.72	0.427	0.927
Clinical-lab	0.8282 (0.8021–0.8544)	0.516	0.813	0.703	0.421	0.934
**Validation cohort**						
Clinical model	0.7163 (0.6702–0.7624)	0.327	0.631	0.696	0.353	0.878
Lab-score	0.8056 (0.7629–0.8483)	0.506	0.631	0.875	0.571	0.9
Clinical-lab	0.8221 (0.7826–0.8615)	0.524	0.738	0.786	0.475	0.919

*AUC, area under the curve; CI, confidence interval; HE, hematoma expansion; NPV, negative predictive value; PPV, positive predictive value*.

### Clinical Use

**Figure 5:**
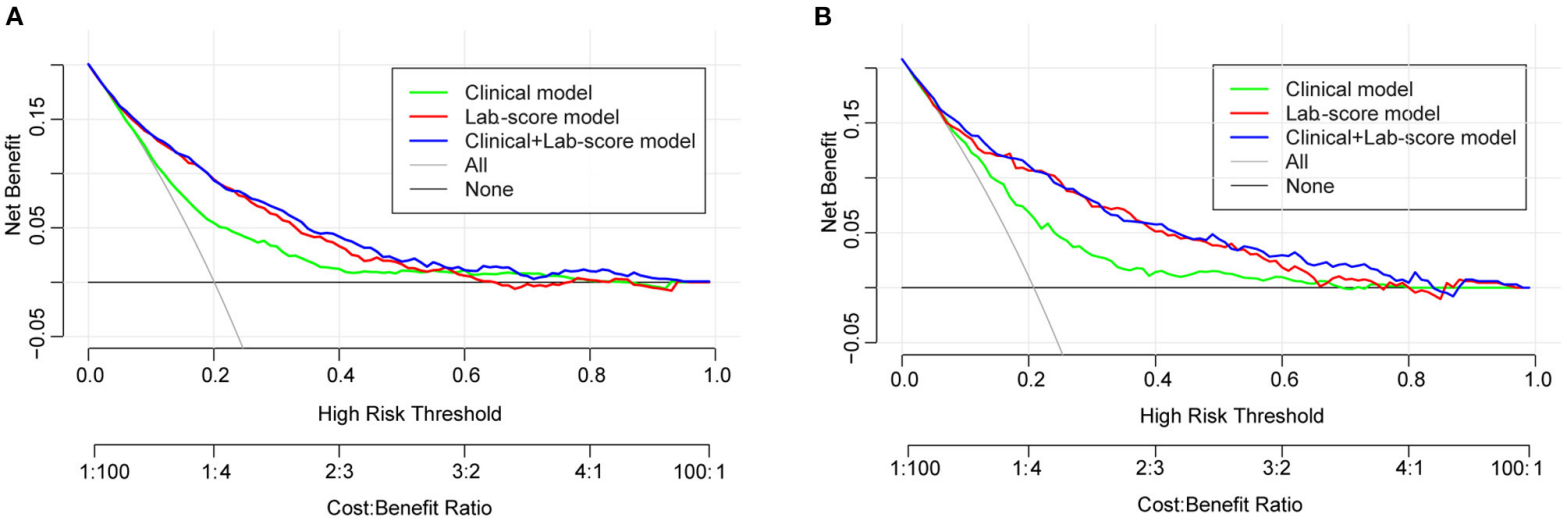
**(A)** Decision curve analysis for the clinical-lab nomogram. The y-axis measures the net benefit. The red line represents the lab-score nomogram. The blue line represents the clinical-lab nomogram. The green line represents the clinical nomogram. Thin black line represents the assumption that no patients have IAID. **(B)** The net benefit was calculated by subtracting the proportion of all patients who are false positive from the proportion who are true positive. The net benefit was comparable, with several overlaps, on the basis of the clinical-lab nomogram and the lab-score nomogram.

The decision curve analysis for the clinical-lab nomogram was presented in Figure 5. If the threshold probability is >5%, using the clinical-lab nomogram to predict IAID added more benefit than the treat-all or the treat-none patients. The decision curve demonstrated that the clinical-lab model added more net benefit than either the lab-score model or the clinical model in differentiating IAID from AIS patients within the range of the threshold probability of 0.05 to 0.8 (Figure 5A). Similar results were observed in the validation dataset, indicating an excellent interobserver agreement of decision curve analysis (Figure 5B).

## Discussion

In the present study, we developed and validated a clinical and laboratory signature-based nomogram for the infectious and inflammatory disorders individualized prediction in patients with acute ischaemic stroke during hospitalization. The nomogram incorporates two parameters of the lab score and clinical risk factors (age, comatose state, diabetes, and CHF) (35, 36). The clinical-lab signature successfully stratified patients according to their risk of infectious and inflammatory disorders. Incorporating the lab-score and clinical signatures into an easy-to-use nomogram facilitates the infectious and inflammatory disorder's individualized prediction of acute ischaemic stroke. DCA showed that the clinical-lab nomogram was clinically valuable. Combining the clinical characteristics and lab-score for predicting the risk of IAID with the inpatient AIS has not been reported to the best of our knowledge.

This study addresses a very significant clinical problem that differentiates IAID from non-IAID in acute ischaemic stroke. It may not be suitable for the AIS population to passively manage the risk of infection and inflammation activation during hospitalization. It is especially true when acute ischaemic stroke patients with a higher probability of having IAID, which is very different from the recovery of neurological function and prognosis. Although laboratory indicators suggest infectious and inflammatory disorders in hospitalized patients, the quantitative prediction seems to be more difficult. Studies of patients with acute stroke have shown that the percentage of neutrophils and NLR may be predictors of infection, while the accuracy ranges from 53 to 75% (37). It highlights the necessity of differentiating the risk of infectious and inflammatory disorders in patients with acute stroke during hospitalization in an accurate and timely manner. Clinically, there is a need for a diagnostic biomarker for AIS, especially in IAID. Other diagnostic biomarkers of infectious or inflammatory disorders such as serum percentage of neutrophil, C-reactive protein, and procalcitonin are non-specific for uropathogens and respiratory pathogens or cannot differentiate between them. The clinical-lab signature in acute ischaemic stroke patients supports the predictive ability of this combined marker for IAID. On the other hand, although the infectious and inflammatory disorders of elderly patients increase the difficulty of clinical treatment, they can also subject patients to the inherent risk other than the primary disease. Furthermore, a conservative approach to watchful waiting may bring about the intermittent progression of acute, curable stroke (35, 36).

Clinical characteristic signatures including age, comatose state, diabetes, and CHF are available on admission; it is of interest whether they are independent risk factors for IAID. Unexpectedly, clinical characteristics alone to the prediction model did not have a satisfactory classification performance (C-index, 0.71, in derivation and validation cohort) (2, 35, 36). On the other hand, the addition of lab-score to the prediction model and characteristic clinical signatures significantly improved the reclassification performance (C-index, 0.83, in derivation cohort; and C-index, 0.82, in the validation cohort). The clinical-lab signature that integrates multiple individual laboratory features in this work showed sufficient discrimination in the derivation dataset, which surprisingly showed favorable results in the validation cohort. Considering that the positive rate of IAID was comparable in the two cohorts, the improved discrimination implies that the clinical-lab signature was robust for prediction. Thus, the easy-to-use clinical-lab nomogram could serve as a more convenient biomarker for the prediction of IAID. Previous single marker studies of AIS have shown that patients with higher C-reactive protein levels had more unfavorable outcomes (AUC, 0.641) (7). Note that the C-reactive protein level did not show enough predictive strength based on univariable. Although studies have shown that the ratio of nomogram neutrophils to lymphocytes can predict stroke-related infections, our work includes constructing and verifying larger sample models, and the results seem to be more accurate (1, 38). Multimarker clinical studies have shown that panel of laboratory biomarkers incorporated male sex, systolic blood pressure, glucose, nitric oxide metabolites, lipid hydroperoxides, 25-hydroxyvitamin D, IL-6, and WBC with the sensitivity of 86.2% and specificity of 93.0% are related to ischemic stroke and predict a poor outcome at 3-month follow-up (39). In a recent study, observing the diagnostic accuracy of lymph node metastasis in colorectal cancer, the training accuracy of the radiomics nomogram was lower than 80% (40). The relatively low accuracy rate may be related to the inherent heterogeneity of the tumors.

For the construction of the clinical-lab signature, 55 candidate laboratory features were reduced to 8 potential predictors by using the LASSO algorithm with 10-fold cross-validation to shrink the regression coefficients. This method surpasses the technique of choosing predictors according to their univariable association strength association with outcome and enables the panel of selected features to be integrated into a clinical-lab signature as well (41). In recent studies, multimarker analyses adding individual markers into marker panels have been widely used in clinical prognosis or risk prediction. For instance, in an inpatient stroke rehabilitation analysis, discharge scores of clinical outcomes could be predicted based on patient demographics and medical information, such as age, sex, body mass index, race, and time from stroke onset to rehabilitation admission (42).

As quantitative peripheral blood routine examination indicators, the percentage of HGB and CRP can be easily obtained. Interestingly, in the current study, lab-score signature relied on percentages but not absolute values of circulating leukocyte populations. One of the reasons may be the previous research results that neutrophil-to-lymphocyte ratio and platelet-to-lymphocyte ratio were weakly correlated with inflammatory biomarkers (43). Accumulating evidence suggests that neutrophils in peripheral blood play essential roles in controlling the invasion of bacteria into the human body and various inflammation-related diseases (44, 45). Elevated neutrophils mean non-specific inflammation (46). The concept is that the amelioration of the inflammatory state is characterized by the decrease of neutrophil and C-reactive protein levels (37). An inflammatory imbalance with changes in circulating leukocytes and C-reactive protein levels has been observed in patients with AIS (37, 47). Meanwhile, inflammation can release hemoglobin or heme from red blood cells (47). Furthermore, existing studies have shown that hemoglobin could serve as an essential indicator of inflammatory response in patients with AIS (47–49); therefore, we kept hemoglobin level as a candidate factor in the process of model development.

The use of a clinical-lab nomogram may need to interpret the individual need for additional treatment or care. Therefore, to justify the clinical application value, we assessed whether the clinical-lab nomogram-assisted decisions would improve patient outcomes. With this aim, this study used decision curve analysis rather than the verification of the nomogram by multi-institutional validation because of the heterogeneity of clinical characteristics and laboratory indicators acquisition in different institutions. Based on the clinical consequences of threshold probability, the net benefit could be derived.

Study limitations include the design as a retrospective study and the lack of National Institutes of Health Stroke Scale (NIHSS) functional outcome data. Another limitation is the underestimation of some acute inflammatory disorders because of the absence of a diagnostic code. In addition, common infections and inflammatory respiratory illnesses are often diagnosed in primary care. Finally, we did not include information on specific indications for antibiotic and anti-inflammatory drugs and data on over-the-counter drugs, which mainly affected the use of NSAIDs.

## Conclusion

This study presents a clinical-lab nomogram that incorporates both the clinical risk factors and lab-score signature and can be conveniently used to facilitate the individualized prediction of infectious and inflammatory disorders in hospitalized patients with acute ischemic stroke (AIS).

## Data Availability Statement

The raw data supporting the conclusions of this article will be made available by the authors, without undue reservation.

## Ethics Statement

The studies involving human participants were reviewed and approved by the Ethics Committee of The First Affiliated Hospital of Guangxi University of Chinese Medicine, 2020-029-01. Written informed consent for participation was not required for this study in accordance with the national legislation and the institutional requirements.

## Author Contributions

JunL and YH: Conceptualization. JunL and ZC: Data curation, visualization, and investigation. JunL: Formal analysis and funding acquisition. LW: Methodology. YH: Project administration. WC: Resources. TP: Software. JunL, JH, and JinL: Writing—original draft. JH, ZC, and WC: Writing—review & editing. All authors approved the final draft of the manuscript for publication.

## Funding

This study was funded by Guangxi Key Research and Development Project (Grant Number: guikeAB18221020), Guangxi Science and Technology Project (Grant Number: 2019GXNSFBA245091), Guangxi Key Laboratory of Chinese Medicine Foundation Research Project (Grant Number: 19-245-14-03), Innovation Project of Guangxi Graduate Education (Grant Number: YCBZ2020067), and Guangxi First-class Discipline Construction Project (Grant Number: 2019XK025 and 2019XK024).

## Conflict of Interest

The authors declare that the research was conducted in the absence of any commercial or financial relationships that could be construed as a potential conflict of interest.

## Publisher's Note

All claims expressed in this article are solely those of the authors and do not necessarily represent those of their affiliated organizations, or those of the publisher, the editors and the reviewers. Any product that may be evaluated in this article, or claim that may be made by its manufacturer, is not guaranteed or endorsed by the publisher.

## Abbreviations

AIS: acute ischemic stroke
CCI: Charlson comorbidity index
CHD: coronary heart disease
CHF: congestive heart failure
COPD: chronic obstructive pulmonary disease
CVD: cerebrovascular diseases
DCA: decision curve analysis
IAID: infectious and inflammatory disorders
LASSO: least absolute shrinkage and selection operator
ROC: operating characteristic.
